# Brainstem Tuberculoma in Pregnancy

**DOI:** 10.1155/2015/179483

**Published:** 2015-11-04

**Authors:** Dana A. Muin, Katrin Wagner, Rosemarie Burian, Naghmeh Ghaem Maghami, Olav Lapaire

**Affiliations:** ^1^Department of Obstetrics and Fetomaternal Medicine, University Hospital Basel, Spitalstrasse 21, 4031 Basel, Switzerland; ^2^Department of Obstetrics and Fetomaternal Medicine, Medical University Vienna, Waehringer Guertel 18-20, 1090 Vienna, Austria

## Abstract

We report a case of a Somali refugee who presented in the second trimester of her first pregnancy with a four-week history of gradual right-sided sensomotoric hemisyndrome including facial palsy and left-sided paresis of the oculomotorius nerve causing drooping of the left eyelid and double vision. Cranial magnetic resonance imaging revealed a solitary brainstem lesion. Upon detection of hilar lymphadenopathy on chest X-ray (CXR), the diagnosis of disseminated tuberculosis with involvement of the central nervous system was confirmed by PCR and treatment induced with rifampicin, isoniazid, pyrazinamide, and ethambutol. The patient had a steady neurological improvement and a favorable pregnancy outcome.

## 1. Background

Tuberculosis (TB) is a contagious airborne disease caused by* Mycobacterium tuberculosis *that predominantly affects poor and vulnerable population groups from low- and middle-income countries that are at increased risk of TB exposure and transmission [1]. In 2013, nine million people became infected with TB of whom 3.3 million (37%) were women. Globally, TB belongs to the top five causes of death in women between 15 and 44 years of age. In 2011, approximately 216.500 women were infected during pregnancy.

With an incidence of 1.5 million deaths in 2013, TB ranks as the second leading cause of death from an infective agent, after human immunodeficiency virus (HIV). Nearly 70% occurred in African, Southeast Asian, and Western Pacific regions. After all, since 1990, there has been a 45% decline in TB mortality and a 41% decline in TB prevalence worldwide. Much of this success is merit of the globally improved diagnostic process and better access to TB care and treatment, all of which have the WHO-goal to end this epidemic.

TB most often affects the pulmonary system and exerts common symptoms such as cough with bloody sputum, chest pains, weakness, weight loss, fever, and night sweats. Rarely, it can disseminate through the body as miliary TB and in 1% of cases it involves the central nervous system. During pregnancy, vertical transmission from an affected mother to her fetus is extremely rare. However, there is a sixfold increase in perinatal death and a twofold risk of premature birth and low birth-weight. Concomitant HIV is even associated with a 300%-fold increased risk of maternal and fetal mortality [2].

TB is a curable disease that is usually treated with a standard 6-month course of four antimicrobial drugs (rifampicin, isoniazid, pyrazinamide, and ethambutol). With proper treatment, an estimated 37 million lives were saved between 2000 and 2013. Up to 2/3 of patients with TB would die from this disease, if they did not receive standard anti-TB drugs. Yet between 2009 and 2013, the number of patients with single- or multidrug resistant tuberculosis (MDR-TB) has tripled and reached approximately 480.000 in 2013. Most commonly, bacteria would fail to respond to isoniazid or rifampicin. The primary cause for MDR-TB is inappropriate treatment or incorrect use or use of poor quality anti-TB drugs. To combat this challenge, new anti-TB drugs are currently under investigation and screening programs in place to detect people who would most likely benefit from new medications.

In the following, we report a case of a solitary brainstem tuberculoma manifesting itself with progressive neurological symptoms. Upon diagnosis and commencement of the WHO-recommended quadruple therapy, the symptoms improved rapidly and the pregnancy was carried out until term with delivery of a healthy male infant.

## 2. Case Presentation

A 19-year-old Somali gravida I para 0 with no significant past medical and obstetric history presented to our antenatal clinic at 28^+1^ weeks of gestation (/40). She had arrived in Switzerland eight days before, following a strenuous political migration from Somalia via Ethiopia, Sudan, Egypt, Libya, Tunisia, and Italy. On this journey lasting for over 12 months, she had felt progressively unwell with weakness of the right side of her body, as well as double vision and a drooping left eyelid. She had occasional events of vertigo and confusion. Clinical examination confirmed the right-sided sensomotoric hemisyndrome with facial palsy and left-sided paresis of the oculomotorius nerve.

## 3. Investigations

**Figure 1:**
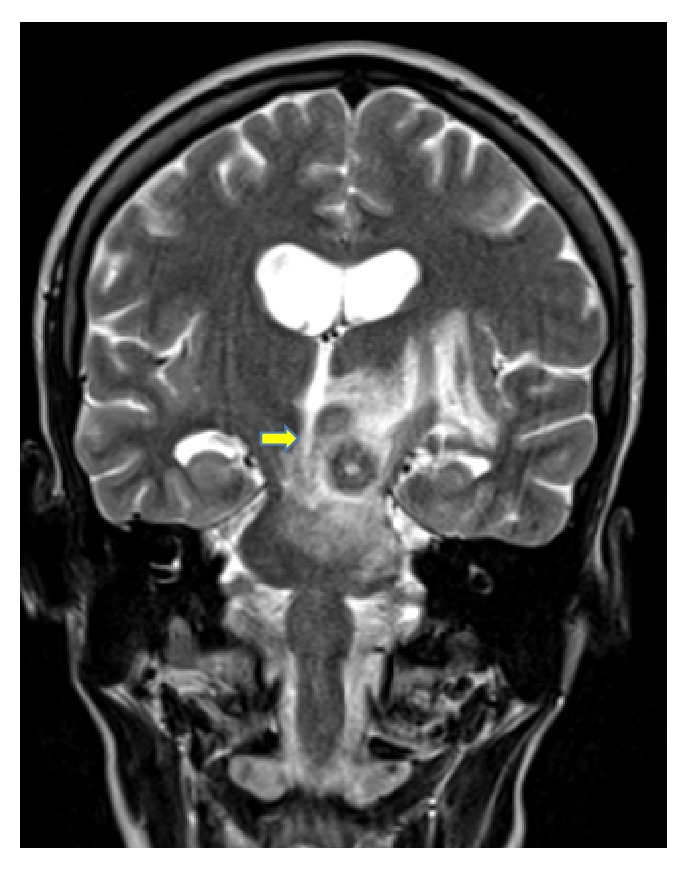
Tuberculoma in the left pedunculus cerebri at time of first encounter (frontal view).

**Figure 2:**
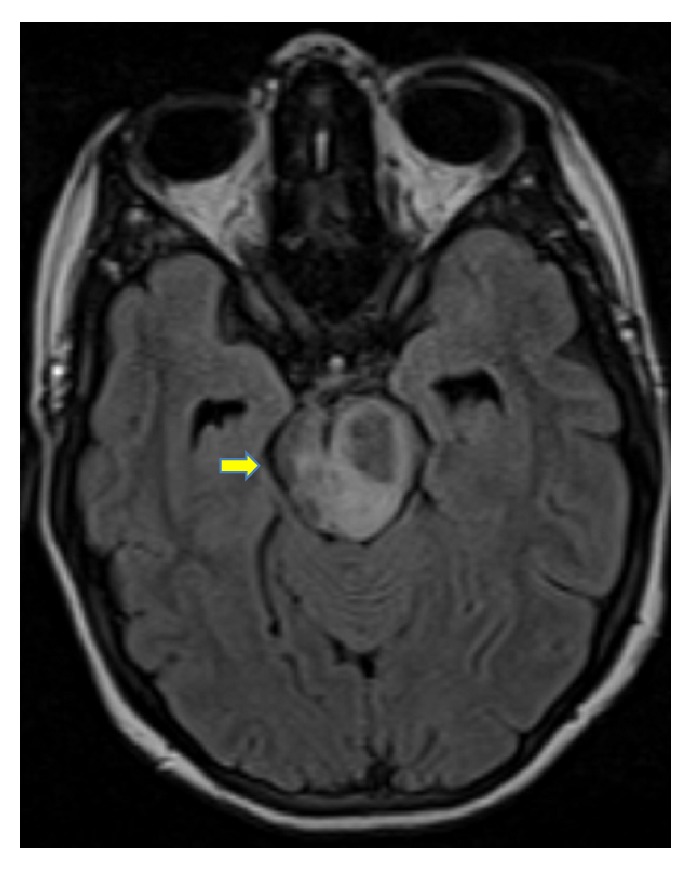
Tuberculoma in the left pedunculus cerebri at time of first encounter (horizontal view).

Upon arrival, the patient underwent cranial magnetic resonance imaging (MRI), which showed an 18 × 12 × 18 mm solitary lesion in the left brainstem with annular enhancing and perifocal edema (Figures 1 and 2). There was concomitant partial compression of the third ventricle and aqueduct. Furthermore slightly dilated side ventricles were noted as sign of compression. This lesion was suggestive of an intracranial tumor or abscess of an infective agent. A cerebrospinal fluid examination was unremarkable and showed a normal cell count and protein level, slightly elevated lactate (2.1 mmol/L; normal range: 1.1–1.9 mmol/L) and glucose (5.0 mmol/L; normal range: 2.2–4.2 mmol/L), and normal lactate/glucose ratio (0.5; normal range: >0.5). Liquor cell pathology was negative for malignant cells and microbiology did not grow any cryptococci,* Mycobacterium tuberculosis*, fungi,* Nocardia*, and* Histoplasma* nor aerobe or anaerobe bacteria.

A TORCH-panel test was performed, which included serology for* toxoplasmosis*,* rubella*,* cytomegalic virus*,* herpes simplex virus types 1 and 2*,* varicella zoster virus*,* lues*,* German measles*,* Epstein-Barr virus*,* human immunodeficiency virus*, and* hepatitis* serology, which all revealed unremarkable results. With a past medical history of* hepatitis B* (HB), the patient's serology was positive for HBs-antibodies and* hepatitis C*-antibodies and negative for HBs-antigen. Quantitative HB count was <20 IU/mL.

**Figure 3:**
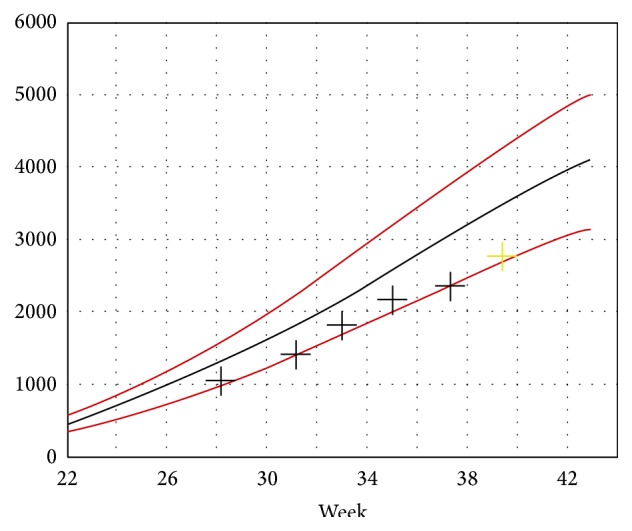
Fetal weight curve.

Upon arrival, an antenatal ultrasound scan was performed, which showed an intact intrauterine singleton pregnancy with restricted growth measurements, which remained along the 3rd percentile throughout pregnancy (Figure 3). There were no sonographic signs of fetal anomalies or polyhydramnion and the scan ruled out signs of fetal toxoplasmosis.

**Figure 4:**
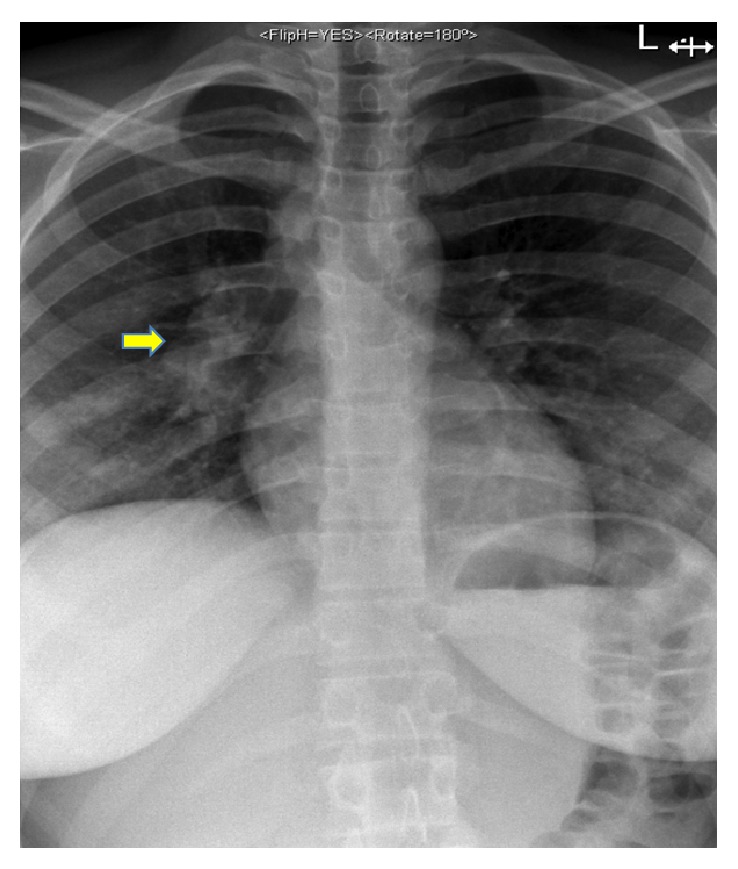
Chest X-ray.

An abdominal ultrasound scan revealed unremarkable liver, kidneys, and spleen and no signs of abscesses or parasitic cysts in the internal organs of the patient. A CXR was performed which revealed a right-sided hilar lymphadenopathy (Figure 4). During a subsequent bronchoscopy, a large infracarinal lymph node was biopsied which was diagnosed as granuloma in histology. A bronchoalveolar lavage retrieved cells, which were identified as* Mycobacterium tuberculosis* in rapid PCR (GeneXpert). Bacterial culture of the same material confirmed the diagnosis of TB and revealed sensitivity to rifampicin, isoniazid, pyrazinamide, ethambutol, and streptomycin.

Although no histological investigation was obtained from the brain lesion itself, the diagnosis was made as open pulmonary tuberculosis with central dissemination and growth of a tuberculoma in the left brainstem leading to neurological deficits in this 19-year-old patient.

## 4. Treatment

The patient was hospitalized and isolated in a single-bed room in the medical ward of our hospital for the first two weeks. A multidisciplinary meeting took place with the local obstetricians, anesthesiologists, neonatologists, endocrinologists, neurologists, and infectious disease specialists in order to define a peri-, intra-, and postpartum management plan. In view of the early gestational week and possible need for early delivery, fetal lung maturation was induced by administration of two standard doses 12 mg intramuscular glucocorticoid injections 24 hours apart. Further corticosteroid treatment was given to the patient in order to reduce the size of the perifocal edema in the brain and eliminate neurological symptoms. On recommendation of the hospital endocrinology team, 5 mg oral prednisone was given once in the morning until delivery. A Synacthen test was performed along this course in order to rule out a secondary adrenal insufficiency and was unremarkable.

According to the WHO-recommendation for TB in pregnancy, the patient was started on a quadruple regimen, consisting of treatment with rifampicin, isoniazid, pyrazinamide, and ethambutol, followed by a 10-month treatment of rifampicin and isoniazid. For better compliance and closer fetomaternal monitoring, the patient was kept hospitalized at our obstetric department from 32^+6^ to 36^+3^/40. During this time, weekly ultrasound scans were performed to monitor fetal growth and ensured fetal wellbeing.

At 40^+5^/40, the patient was readmitted to our clinic for induction of labor due to postdates and oligohydramnion with an amniotic fluid index of 2.7. The patient received 100 mcg oral misoprostol, 25 mg oral hydrocortisone, and adequate saline hydration intravenously every six hours. In the first stage of labor, Syntocinon infusion was commenced at 3 cm cervical dilation to support contractions. Upon amniotomy at 4 cm dilatation, meconium stained liquor was drained. In view of a suspicious CTG with variable decelerations and failure to progress for 190 minutes, the decision was eventually taken for a secondary caesarean section (Robson Classification 2a). A slightly depressed male newborn was delivered from the first occipitoposterior cephalic position (APGAR 6/10/10; weight 2710 g (below 3rd percentile), body length 45 cm, and head circumference 33 cm; pH umbilical artery 7.28, pH umbilical vein 7.35). Neonatal examination showed no signs of infection in the newborn or any anomalies.

Postpartum, the corticosteroid dose was tapered down to Solu-Cortef 50 mg i.v. every 12 hours on day one, followed by Solu-Cortef 50 mg i.v. once on day two and hydrocortisone 10 mg twice on days three (3-1-0) and four (1-1-0).

## 5. Outcome and Follow-Up

**Figure 5:**
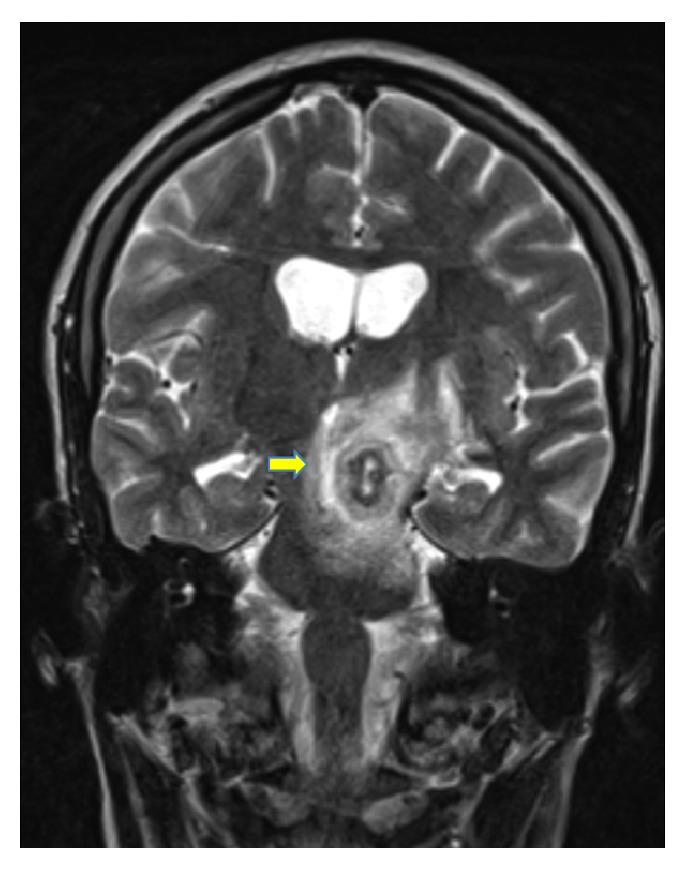
Tuberculoma after 1 month (frontal view).

**Figure 6:**
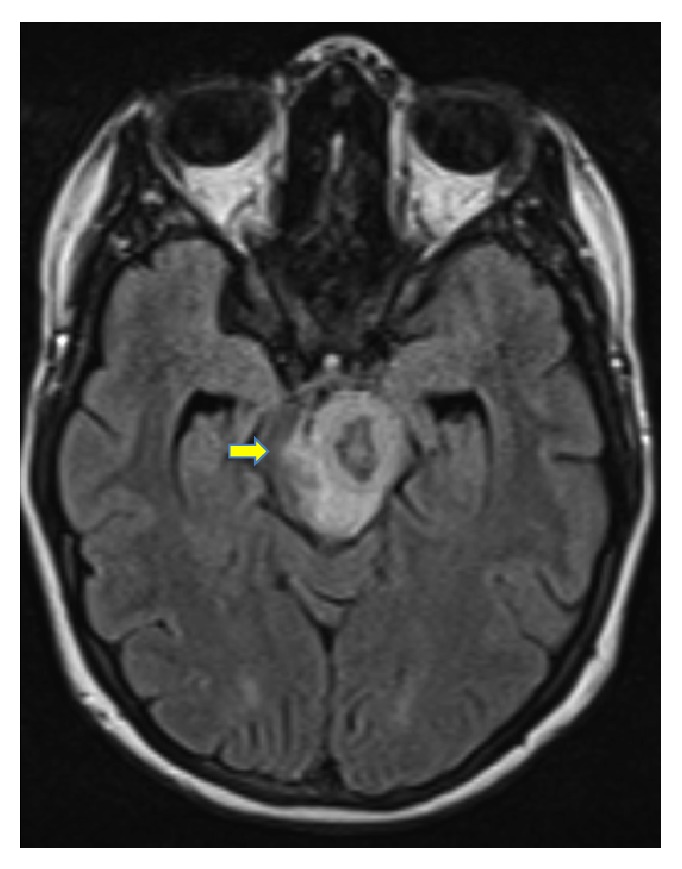
Tuberculoma after 1 month (horizontal view).

**Figure 7:**
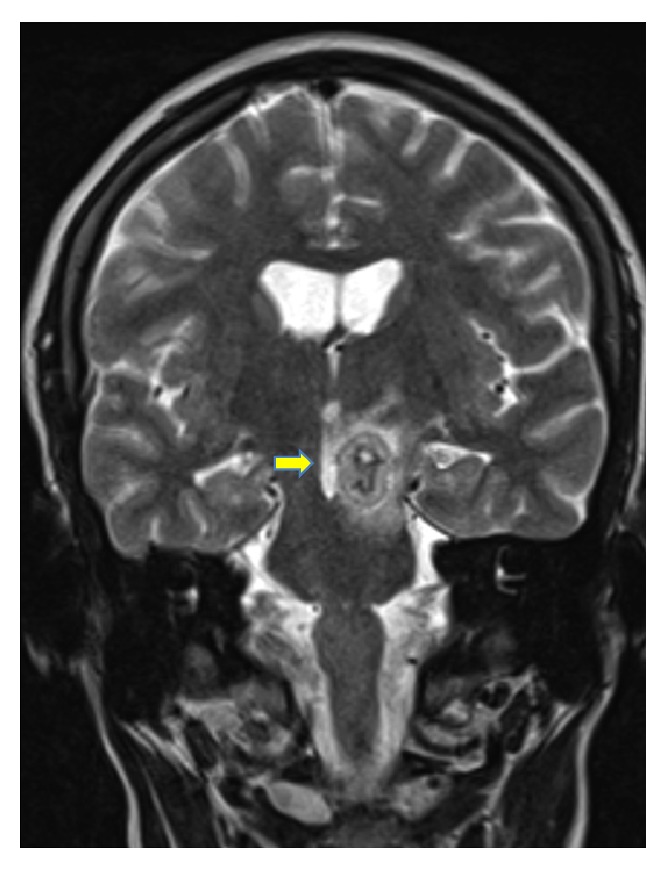
Tuberculoma after 2 months (frontal view).

**Figure 8:**
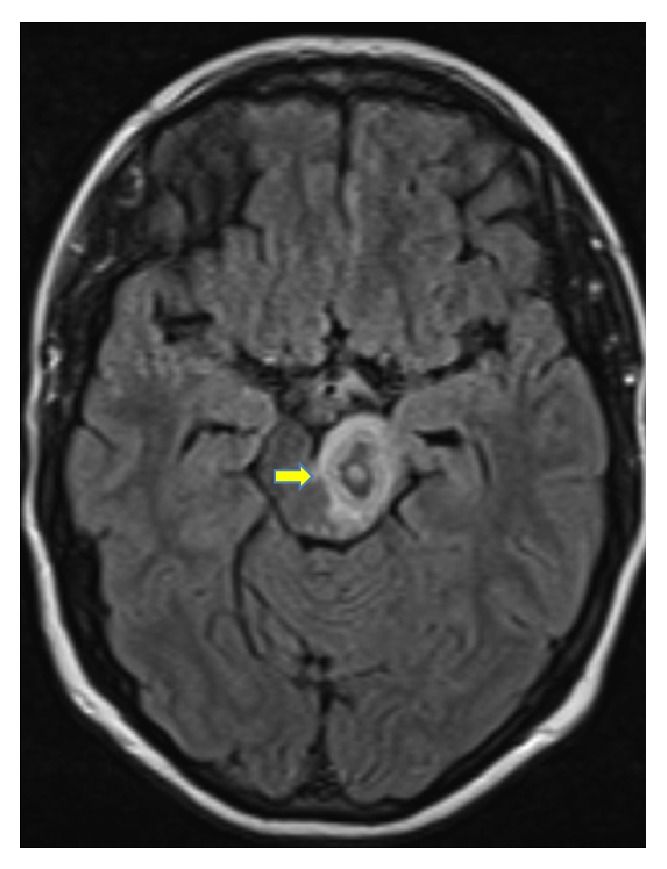
Tuberculoma after 2 months (horizontal view).

During pregnancy, the patient underwent follow-up cranial MRI one and two months after induction of anti-TB treatment. Figures 5 and 6 illustrate a reduction of the perifocal edema around the tuberculoma in the left pedunculus cerebri in comparison to previous results. The lesion itself showed a reduction in its size (15.8 mm from 18 mm). Around the hypointense ring enhancement, there was a hyperintense to mixed intense ring enhancement. Furthermore a remaining shift and compression of the third ventricle were noted with no progression of dilatation of the ventricular system. Two months afterwards, the perifocal edema continued to show a regression in its size and extent (Figures 7 and 8) with a smaller sized tuberculoma in the left pedunculus cerebri (13.3 mm).

Clinical examination showed improving neurological symptoms and the oculomotorius paresis completely resolved within two months of treatment. A control sputum examination was performed three months after treatment and showed no further growth of the* Mycobacterium tuberculosis*. Postpartum, control-MRI was arranged one month after delivery, which the patient unfortunately did not attend.

## 6. Discussion 

*Mycobacterium tuberculosis* is an aerobic, acid-fast, nonmotile, nonencapsulated bacillus, whose transmission is usually via inhalation. Intracerebral tuberculosis constitutes approximately 5–30% of all intracranial lesions causing (spinal) meningitis, intracerebral or spinal tuberculoma, tubercular abscess meningitis, encephalopathy, or vasculopathy, especially in immunocompromised patients [3, 4]. Common locations of tuberculomas are the corticomedullary junction and periventricular region that are reached via the hematogenous route. In 15–34% of cases, intracranial tuberculomas occur multiply and can mimic secondary blastomatous disease in the brain causing intracranial pressure with headache, papilledema, focal neurological deficits like hemiparesis, vertigo, ataxia, and seizures, or behavioral changes [5–7].

On microscopy, tuberculomas have a thick fibrous wall with chronic granulomatous inflammation bearing* Langhans*-type giant cells and central caseating necrosis with perilesional secondary granulomatous vasculitis that may cause occlusion of the vessel lumen and making penetration of anti-TB drugs more difficult.

In pregnancy, the T-helper 1 (Th1) proinflammatory response is suppressed, which increases susceptibility to new infections and reactivation of TB, while, after delivery, Th1 suppression reverses, thus exacerbating symptoms and making women develop TB twice as likely in the early postpartum phase compared to outside pregnancy [8–10].

Gynecological complications of TB outside pregnancy include salpingitis, endometritis, cervicitis, and irregular vaginal bleeding. In pregnancy, TB is not known to be teratogenic; however increased perinatal mortality and intrauterine growth restriction are very common and affect 66% of pregnancies of TB-infected mothers [11, 12]. Reasons for this may be placental infection and insufficiency, maternal malnutrition, anemia, and antenatal poor nutritional status.

Intrapartum standard treatment of TB with isoniazid, rifampicin, pyrazinamide, and ethambutol for 2-3 months, followed by isoniazid and rifampicin for further 12–15 months, has a good safety record in pregnancy [13, 14]. First-line drugs in intrapartum treatment of tuberculosis include isoniazid (Food and Drug Administration Pregnancy Category A), which can also be administered as chemoprophylaxis in latent TB. In pregnant women, isoniazid may be associated with hepatotoxicity; therefore liver function tests should be performed in the pregnant woman every two weeks during the first two months of treatment and monthly thereafter. Isoniazid is also associated with peripheral neuropathy, of which pregnant women are at increased risk; hence prophylactic pyridoxine treatment is recommended in pregnancy.

Rifampicin (Food and Drug Administration Pregnancy Category C) eliminates low or nonreplicating organisms and is associated with hemorrhagic disease in newborns due to hypoprothrombinemia. Vitamin K should therefore be given to newborns whose mothers were on rifampicin during pregnancy [15, 16].

Ethambutol (Food and Drug Administration Pregnancy Category A) eliminates most of the rapidly proliferating bacilli in the first 14 days of treatment. Side effects in adults may include retrobulbar neuritis after high doses, although no adverse fetal outcome has been reported [15, 16].

Pyrazinamide (Food and Drug Administration Pregnancy Category n/a) kills bacilli with low-penetration site for other drugs. To date, there are no reports of significant adverse events from the use of this anti-TB medication during pregnancy [17].

Here, we presented the case of a brainstem tuberculoma that led to significant neurological impairment. Our differential diagnosis included rhombencephalitis by* Listeria monocytogenes*, sarcoidosis, and an intracranial process of benign or malignant pathology (e.g., glioma, neurological lymphoma). Radiological imaging suggested toxoplasmosis or cerebral TB. With regard to the pathogenesis, we ruled out other bacterial infections in the central nervous system (e.g.,* Listeria*,* Brucella*, and* Nocardia*), an intracerebral abscess (e.g., bacterial transmission from the oropharyngeal region or sinuses), and intracranial parasitic (*toxoplasmosis*,* neurocysticercosis*,* Echinococcus*,* Entamoeba histolytica*,* schistosomiasis*,* Paragonimus*, and* leishmaniosis*), viral (*herpes simplex*), or fungal (*Cryptococcus*,* histoplasmosis*, and* coccidiomycosis*) infections. Once TB was confirmed by PCR and the standard therapy for TB in pregnancy commenced without delay, we witnessed rapid improvement in this 19-year-old Somali with satisfactory maternal and fetal outcome.

With the steadily increasing rate of refugees from the most vulnerable African populations, a high prevalence of TB-affected pregnant patients is to be expected in Europe in the coming years. It is therefore recommendable to get familiarized with international recommendations for intrapartum treatment of TB as well as associated risk factors of TB in and outside pregnancy.

## 7. Learning Points/Take-Home Messages

1. Beware of the risk factors for tuberculosis in patients with migration background and think of brainstem dissemination if neurological symptoms present.
2. Early diagnosis of tuberculosis is necessary to provide good fetomaternal care and outcome.
3. Aim for interdisciplinary treatment in pregnant women with tuberculosis.

## 8. Conclusion

By this case report we wanted to show the rare manifestation of central dissemination of pulmonary tuberculosis involving the brainstem mimicking signs and symptoms of an intracerebral tumor. The patient's symptoms resolved gradually yet completely within three months of anti-TB treatment. High index of suspicion and timely intervention are required to diagnose and deal with this potential fatal but easily treatable condition.

Untreated tuberculosis in pregnancy has a high risk for both mother and fetus and should therefore be dealt with in a specialist center where a multidisciplinary approach is available and neonatal care provided in case of preterm delivery. Regular follow-up visits are important for early detection of fetal growth restriction and maternal side effects from treatment.

This case report confirms the positive risk-benefit profile of the quadruple anti-TB regimen in pregnancy.
